# The Oxidation of Equol by Tyrosinase Produces a Unique Di-*ortho*-Quinone: Possible Implications for Melanocyte Toxicity

**DOI:** 10.3390/ijms22179145

**Published:** 2021-08-24

**Authors:** Hitomi Tanaka, Shosuke Ito, Makoto Ojika, Tomoko Nishimaki-Mogami, Kazunari Kondo, Kazumasa Wakamatsu

**Affiliations:** 1 Department of Medical Technology, School of Health Sciences, Gifu University of Medical Science, 795-1 Nagamine, Ichihiraga, Seki 510-3892, Japan; hitanaka@u-gifu-ms.ac.jp; 2 Institute for Melanin Chemistry, Fujita Health University, 1-98 Dengakugakubo, Kutsukake-cho, Toyoake 470-1192, Japan; sito@fujita-hu.ac.jp; 3 Graduate School of Bioagricultural Sciences, Nagoya University, Chikusa-ku, Nagoya 464-8601, Japan; ojika@agr.nagoya-u.ac.jp; 4 Division of Biochemistry, National Institute of Health Sciences, Kawasaki-ku, Kawasaki 210-9501, Japan; mogami@nihs.go.jp (T.N.-M.); kondo@nihs.go.jp (K.K.)

**Keywords:** anti-aging, antioxidant, melanocyte toxicity, *ortho*-quinone, equol

## Abstract

Equol (7-hydroxy-3-(4′-hydroxyphenyl)-chroman, EQ), one of the major intestinally derived metabolites of daidzein, the principal isoflavane found in soybeans and most soy foods, has recently attracted increased interest as a health-beneficial compound for estrogen-dependent diseases. However, based on its structure with two *p*-substituted phenols, this study aimed to examine whether EQ is a substrate for tyrosinase and whether it produces *o*-quinone metabolites that are highly cytotoxic to melanocyte. First, the tyrosinase-catalyzed oxidation of EQ was performed, which yielded three EQ-quinones. They were identified after being reduced to their corresponding catechols with NaBH_4_ or L-ascorbic acid. The binding of the EQ-quinones to *N*-acetyl-L-cysteine (NAC), glutathione (GSH), and bovine serum albumin via their cysteine residues was then examined. NAC and GSH afforded two mono-adducts and one di-adduct, which were identified by NMR and MS analysis. It was also found that EQ was oxidized to EQ-di-quinone in cells expressing human tyrosinase. Finally, it was confirmed that the EQ-oligomer, the EQ oxidation product, exerted potent pro-oxidant activity by oxidizing GSH to the oxidized GSSG and concomitantly producing H_2_O_2_. These results suggest that EQ-quinones could be cytotoxic to melanocytes due to their binding to cellular proteins.

## 1. Introduction

**Figure 1:**
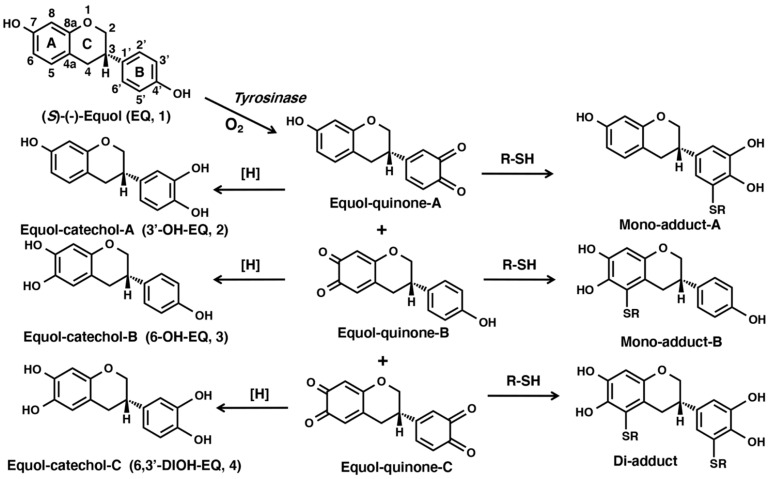
Scheme showing the tyrosinase-catalyzed oxidation of (*S*)-(−)-equol (EQ, **1**) in the absence or presence of a thiol. The oxidation of (*S*)-(−)-EQ **1** gives EQ-quinone-A and -B as immediate products, which are then oxidized to EQ-quinone-C. EQ-quinones are reduced by NaBH_4_ or L-ascorbic acid (AA) to form EQ-catechols, as shown by [H]. The tyrosinase-catalyzed oxidation of (*S*)-(−)-EQ **1** in the presence of a thiol, NAC, CySH, or GSH affords two mono-adducts and one di-adduct.

Equol (7-hydroxy-3-(4′-hydroxyphenyl)-chroman, EQ **1**) has recently attracted increased interest as a health-beneficial compound for estrogen-dependent diseases [1,2]. Equol 1 was the first isoflavanoid identified from fluids extracted from the urine of pregnant mares in 1932 [3]. In 1982, EQ **1** was first detected in human urine and blood as a non-steroidal estrogenic compound [4] and was found in high concentrations in the urine of about 40% of adults who consumed soy foods [5]. EQ **1** is one of the main metabolites of daidzein and is an isoflavane phenolic compound with a non-planar structure that is optically active with an asymmetric carbon atom at the C3 position giving rise to (*R*)-(+)- and (*S*)-(−)-EQ enantiomers (Figure 1). Only (*S*)-(−)-EQ **1** has been detected as a product of bacterial daidzein conversion [6,7,8]. EQ **1** shows an anti-androgenic activity by binding to and sequestering 5α-dihydrotestosterone [9]. In addition, EQ **1** has been shown to have antioxidant activity [10,11] and binds to estrogen receptors to show a stronger estrogenic activity than that of any other isoflavone or isoflavone-derived metabolite [8,12].

Rhododendrol (4-(4-hydroxyphenyl)-2-butanol, RD) is a skin-whitening ingredient that was added to cosmetics by a cosmetic company in Japan. In July 2013, cosmetics containing RD were recalled because a considerable number of consumers developed leukoderma on their faces, necks, and hands [13]. RD was shown to exert melanocyte toxicity via a tyrosinase-dependent mechanism [14]. Ito et al. [15,16] reported that the oxidation of RD by mushroom tyrosinase produced RD-quinone as an intermediate product, which leads to the binding of cellular thiol proteins as well as non-protein thiols, glutathione (GSH), and cysteine (CySH). Furthermore, Ito et al. [16,17] demonstrated that the RD-oligomer derived from RD-quinone exerts a potent pro-oxidant activity by oxidizing GSH and other cellular antioxidants and by concomitantly producing H_2_O_2_.

*trans*-Resveratrol (3,5,4′-trihydroxy-*trans*-stilbene, RES) is a naturally occurring polyphenol that is well known for its antioxidant, antiplatelet, anti-inflammatory, anti-aging, anti-cancer, anti-diabetic, cardioprotective, and cancer chemopreventive properties as well as its neuroprotective properties [18,19]. RES is generally considered a good inhibitor of tyrosinase rather than a substrate [20]. For example, Park et al. showed that RES strongly inhibits mushroom tyrosinase [21]. RES has a *p*-substituted phenol structure similar to that of RD and was found to be a good substrate for tyrosinase and was oxidized to produce a highly reactive *o*-quinone form [22]. This RES-quinone decayed rapidly to produce an oligomer that exhibited a pro-oxidant activity [22]. Based on these results, Ito et al. [22] suggested that the cosmetic use of RES should be considered with caution. Indeed, toxicity and adverse effects were reported following the consumption of RES. Therefore, extensive future studies on the long-term effects, as well as the in vivo adverse effects, of RES supplementation in humans are needed [23].

As mentioned above, the various beneficial health properties of EQ **1** against a variety of disorders, including heart and vascular diseases, osteoporosis, and hormone-dependent cancers (such as those of the breast and prostate) have also been reported [24,25,26]. However, EQ **1** has the same *p*-substituted phenol structure as that of RD and RES. EQ **1** also has a bicyclic chroman structure similar to a metabolic intermediate of RD, RD-cyclic catechol [27]. Thus, EQ **1** is expected to have chemical and biochemical properties similar to those of RD and RES. Nevertheless, it has not yet been reported whether EQ **1** acts as a substrate for tyrosinase as does RD and RES. However, since tyrosinase has recently been adopted for efficient isoflavone hydroxylation in recombinant strains [28] and EQ **1** has a unique structure that has two phenolic groups, the oxidation of EQ **1** by tyrosinase is expected to give rise to EQ-quinone-A and -B as intermediates, which are then oxidized to EQ-quinone-C (Figure 1). These EQ-quinones can be identified after being reduced with NaBH_4_ or L-ascorbic acid (AA) to their corresponding catechols, as was performed to identify the *o*-quinones of RD and RES [22,27]. These EQ-quinones are also expected to react with *N*-acetyl-L-cysteine (NAC), CySH, GSH, and bovine serum albumin (BSA) through their CySH residues to form two mono-adducts and one di-adduct. 

In order to determine whether EQ **1** is a substrate for tyrosinase and whether it produces toxic *o*-quinone metabolites, EQ **1** was oxidized with tyrosinase, and it was determined whether EQ **1** binds to small and protein thiols. Then, the metabolism of EQ **1** was also examined in tyrosinase-transfected cells. Finally, it was shown that the EQ oxidation product, the EQ-oligomer, derived from EQ-quinone, exerts potent pro-oxidant activity by oxidizing GSH to the oxidized glutathione (GSSG) and concomitantly producing H_2_O_2_.

## 2. Results

### 2.1. Tyrosinase-Catalyzed Oxidation of EQ **1** Produces EQ-Quinones

**Figure 2:**
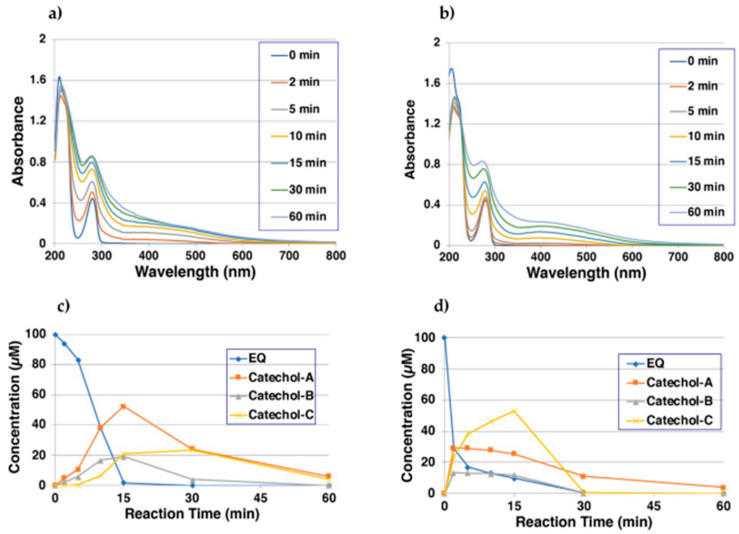
Time course of the tyrosinase-catalyzed oxidation of EQ **1** and HPLC analyses of reaction products. (**a**) UV/visible spectral changes of EQ **1** at pH 6.8; (**b**) UV/visible spectral changes of EQ **1** at pH 5.3; (**c**) HPLC analysis following the tyrosinase-catalyzed oxidation of EQ **1** at pH 5.3, the reaction being stopped by the addition of NaBH_4_, followed by HClO_4_; (**d**) HPLC analysis following the tyrosinase-catalyzed oxidation of EQ **1** at pH 5.3 in the presence of 10 mol eq. AA, the reaction being stopped by the addition of HClO_4_. Data for (**a**,**b**) were obtained from single experiments, but reproducibility was confirmed for each experiment. Data for (**c**,**d**) were obtained from averages of two independent experiments.

The oxidation of 100 µM EQ **1** by mushroom tyrosinase (50 U/mL) was carried out at 37 °C in 50 mM sodium phosphate buffer at pH 6.8. UV/visible spectral changes were followed for 60 min, which showed the rapid production of a quinoid chromophore with absorption at around 400–450 nm (Figure 2a). To slow down the reaction in order to determine the absorption maximum, the oxidation was then carried out at pH 5.3 [22,29], which showed a clear absorption maximum at 420 nm after 10–30 min reaction (Figure 2b).

As the UV/visible spectral changes appeared to be complex, the oxidation at pH 5.3 using high-performance liquid chromatography (HPLC) was performed. As most of the products appeared to be unstable quinones, they were converted to more stable catechols by reducing them with 10% NaBH_4_. As shown in Figure 2c, the oxidation did not proceed quickly at the beginning but then started to proceed faster. EQ **1** was consumed within 15 min, giving new compounds with retention times in HPLC of 7.0, 9.9, and 12.0 min (EQ **1** appeared at 19.4 min). The three EQ-catechols were given the names EQ-catechol-A **2**, EQ-catechol-B **3**, and EQ-catechol-C **4** according to their decreasing order of retention times. The production of three EQ-catechols was expected for the two possible mono-catechol isomers and the one possible di-catechol. The isolation and identification of those three EQ-catechols is described later.

The production of the three possible EQ-catechols during the tyrosinase-catalyzed oxidation was further confirmed by oxidizing 100 µM EQ **1** in the presence of 10 mol eq. AA (1000 µM) at pH 5.3. As shown in Figure 2d, HPLC analysis of the reaction mixtures showed a rapid decrease in EQ **1** in 2 min, giving the three EQ-catechols, which were then oxidized to EQ-quinones after all of the AA was consumed through a redox exchange after ca. 30 min reaction. The time course shown in Figure 2d indicates that EQ-catechol-C **4** was produced from EQ-catechol-A **2** and EQ-catechol-B **3**, which suggests that EQ-catechol-C **4** is most likely a di-catechol.

To confirm the structure of those three EQ-catechols, they were prepared by the tyrosinase-catalyzed oxidation of 1 mM EQ **1** in the presence of 10 mol eq. AA at pH 6.8 for 20 min at 37 °C [22]. The three compounds were assigned as 3′-hydroxy-EQ **2** (EQ-catechol-A; 25% yield), 6-hydroxy-EQ **3** (EQ-catechol-B; 12% yield), and 6,3′-dihydroxy-EQ **4** (EQ-catechol-C; 25% yield) on the basis of ^1^H nuclear magnetic resonance (^1^H-NMR) and high-resolution electrospray ionization (ESI)-time of flight mass spectrometry (TOF MS) spectra. The assignment of the structures of 3′-hydroxy-EQ **2** with a mono-hydroxy group at the 3′ position in the B-ring of EQ **1**, 6-hydroxy-EQ **3** with a mono-hydroxy group at the 6 position in the A-ring of EQ **1,** and 6,3′-dihydroxy-EQ **4** with di-hydroxy groups at the 6 and 3′ positions in the A- and B-rings of EQ **1** was carried out via a comparison with the structural data of EQ **1** previously reported [30,31,32,33] (Figure 1, Figure S1a–d, and Table S1).

### 2.2. Reaction of EQ-Quinones with Non-Protein Thiol Compounds Produces Mono- and Di-Adducts

**Figure 3:**
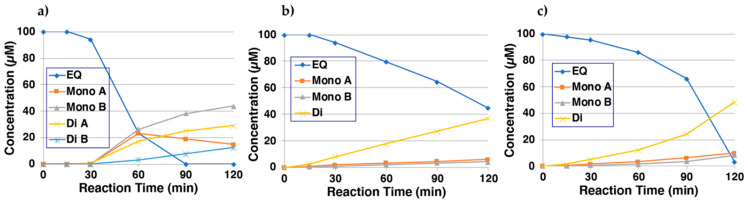
Time course of the tyrosinase-catalyzed oxidation of EQ **1** in the presence of NAC, CySH, or GSH. (**a**) HPLC analysis following the tyrosinase-catalyzed oxidation of EQ **1** in the presence of 3 mol eq. NAC at pH 6.8; (**b**) HPLC analysis following the tyrosinase-catalyzed oxidation of EQ **1** in the presence of 3 mol eq. CySH at pH 6.8; (**c**) HPLC analysis following the tyrosinase-catalyzed oxidation of EQ **1** in the presence of 3 mol eq. GSH at pH 6.8. The reaction was stopped by the addition of HClO_4_. Data were obtained from averages of two independent experiments. Mono-A, mono-B, di-A, and di-B had retention times with decreasing order in each thiol adduct.

Next, it was investigated whether EQ-quinone(s) could bind to thiol compounds [15,22,27,29]. NAC was selected as a model of biologically important thiol compounds. NAC adducts can be extracted with organic solvents and are easily characterized by NMR and MS. Then, 100 µM EQ **1** was oxidized with tyrosinase (50 U/mL) in the presence of 300 µM NAC at pH 6.8. HPLC analysis showed little reaction until 30 min, after which the reaction began and produced four major compounds with retention times of 6.1, 6.6, 7.1, and 7.7 min for two di- and two mono-adducts, respectively (EQ **1** appeared at 19.7 min; Figure 3a). The assignment of di- and mono-adducts is tentative based on the order of retention times; compounds with more thiol addition usually have shorter retention times for hydrophobic catechols [22,29]. Structural assignments of those compounds are described later.

We then examined how CySH and GSH react with EQ-quinone(s). In order to do this, 100 µM EQ **1** was oxidized with tyrosinase in the presence of 3 mol eq. CySH or GSH at pH 6.8. The reaction with CySH (Figure 3b) produced three major compounds at 5.9, 6.6, and 6.9 min. They were tentatively assigned one di-adduct and two mono-adducts. Similarly, the reaction with GSH (Figure 3c) showed the production of three major compounds at 5.8, 6.2, and 6.4 min. They were tentatively assigned one di-adduct and two mono-adducts.

To confirm the structures of the NAC adducts, tyrosinase-catalyzed oxidation of EQ **1** (1 mM) was carried out in the presence of NAC. However, the reaction did not proceed for several h because of the inhibition of tyrosinase activity by thiols. Therefore, 3′-hydroxy-EQ **2** (100 µM) was added as a catalyst to accelerate the oxidation, which then proceeded rapidly and produced two major compounds, which were isolated by preparative HPLC. ^1^H NMR analysis of the product with the longer retention time gave two *meta*-oriented aromatic protons on one benzene ring and three 1,2,4-oriented protons on another benzene ring, along with one NAC moiety (Figures S3a and S4a, and Table S1). Mass data indicated the structure of the mono-adduct of NAC (see Section 4). ^1^H NMR analysis of the product with a shorter retention time gave an isolated aromatic proton and two 1,3-oriented aromatic protons, along with two NAC moieties (Figures S3b and S4b, and Table S1). Mass data indicated the structure of di-adducts of NAC (see Section 4). Based on those NMR and MS data, the structures 5′-monoNAC-3′-hydroxy-EQ **5** (12%) and 5,5′-diNAC-6,3′-dihydroxy-EQ **6** (5%) were assigned to the adducts with a longer and a shorter retention time, respectively (Figure 1). Those mono-adduct and di-adducts that were identified to correspond to Mono-A and Di-A, respectively, are shown in Figure 3a. The structure of Mono-B could not be determined because it had a low yield. It was tentatively assigned as a mono-adduct, but the production of Mono-B increased continuously, so it could be a di-adduct.

The isolation of CySH adducts turned out to be difficult because of their instability. On the other hand, GSH adducts could be isolated using preparative HPLC. 3′-Hydroxy-EQ **2** was used as a source of EQ-quinone. Two GSH adducts were isolated and were subjected to NMR and MS analyses (see Section 4). A slower-eluting adduct was identified as 5′-monoGS-3′-EQ **7** (59%; Figure 1, Figures S5a,b, S5d–f and S6a, and Table S2) and a faster-eluting adduct was 5,5′-diGS-6,3′-dihydroxy-EQ **8** (3%) (Figures S5c and S6b).

### 2.3. Reaction of EQ-Quinones with BSA

**Figure 4:**
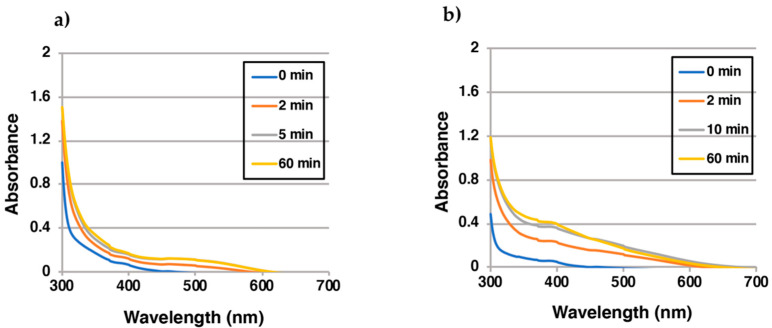
Time course of the tyrosinase-catalyzed oxidation of 3’-hydroxy-EQ **2** in the presence of BSA. (**a**) UV/visible spectral changes of 3’-hydroxy-EQ **2** in the presence of 3 mol eq. BSA (containing 0.90 mol eq. SH group) at pH 6.8; (**b**) UV/visible spectral changes of 3’-hydroxy-EQ **2** in the presence of 3 mol eq. NEM-modified BSA at pH 6.8. Data were obtained from single experiments, but reproducibility was confirmed for each experiment.

In addition, we examined how EQ-quinone reacted with BSA. BSA, which contains one CySH residue in addition to 17 cystine residues, was selected as a representative thiol protein because it is known to react with various o-quinones [15,29,34]. To confirm the possible involvement of CySH in the binding of BSA to EQ-quinone, 100 µM 3′-hydroxy-EQ **2** was oxidized by tyrosinase in the presence of 3 mol eq. *N*-ethylmaleimide (NEM)-modified BSA in which the CySH residue was modified with NEM. The NEM-BSA that reacted with EQ-quinone(s) with an absorption of 0.39 at 400 nm was approximately 2.3 times greater than that with native BSA with an absorption of 0.16 (Figure 4) and also greater than that without protein added (0.23 at 400 nm; Figure 1). The production of a quinoid chromophore between the amino groups of NEM-BSA and EQ-quinone may have caused this large absorption from NEM-modified BSA. These results indicate that the majority of EQ-quinones can bind to BSA through CySH residues as in the case of RES-quinone. [22].

### 2.4. Metabolism of EQ **1** in Tyrosinase-Transfected Cells

**Figure 5:**
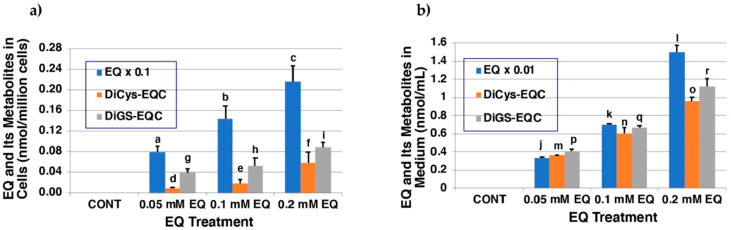
Metabolism of EQ **1** in cells transfected with human tyrosinase yielding di-adducts of CySH and GSH (**a**) in the cells; (**b**) in the medium. The data are mean ± SD from triplicate dishes. Statistically significant differences: *p* < 0.05 (a–b; b–c; d–f; e–f; h–i), *p* < 0.01 (a–c; g–i; n–o), *p* < 0.001 (j–k; k–l; j–l; m–n; m–o; p–q; q–r; p–r). Non-significant differences: g–h (*p* > 0.1); d–e (*p* > 0.05). The statistical significance of the differences was determined by Student’s *t*-test (two-tailed).

We next investigated whether EQ **1** is in fact oxidized to EQ-quinones in cells expressing human tyrosinase, and whether the binding of EQ-quinone to cellular thiols occurs. Ectopic expressions of the human tyrosinase gene in non-melanogenic cells have been reported [35,36]. The human tyrosinase gene in 293T cells, which are widely used for high-level expression of proteins from plasmid vectors, was transiently transfected. Twenty-four hours after transfection, the cells were exposed to EQ **1** for 2 h. The 0.2 mM concentration of EQ **1** is the maximum non-toxic dose. As shown in Figure 5a, EQ **1** was taken up into the tyrosinase-transfected T293 cells and was metabolized in a dose-dependent manner to DiCys-EQ-catechol and DiGS-EQ-catechol. Those di-adducts were the major metabolites, while mono-adducts were only minor products according to the HPLC analysis. DiGS-EQ-catechol was identified as 5,5′-diGS-6,3′-dihydroxy-EQ **8** (Figure 1, Figures S5c and S6b). Those metabolites were then released into the medium (Figure 5b). The EQ **1** content in medium exposed to 100 µM EQ **1** was 70 µM, which contained 0.60 µM DiCys-EQ-catechol and 0.66 µM DiGS-EQ-catechol **8**. Thus, about 2% of the EQ **1** present in the medium was metabolized in cells and released to the medium as Cys and GSH adducts during the 2 h incubation period. Notably, the adducts that formed in the tyrosinase-transfected cells are di-adducts but not mono-adducts. This is consistent with the predominant production of di-adducts in the biochemical experiments (Figure 3b,c).

### 2.5. EQ-Oligomer Oxidizes GSH to GSSG (Pro-Oxidant Activity)

**Figure 6:**
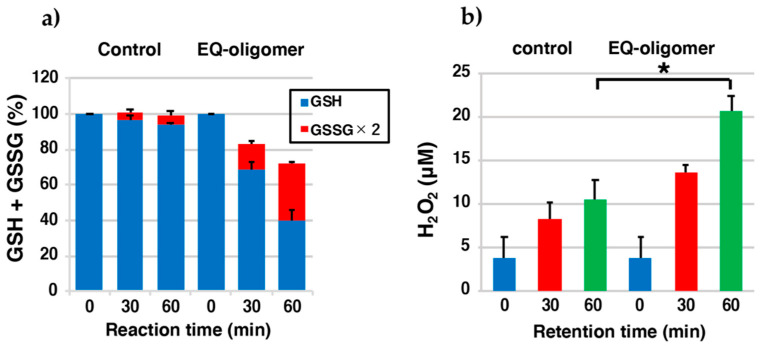
Oxidation of GSH and production of H_2_O_2_ by the EQ-oligomer. (**a**) The consumption of GSH and the production of GSSG from 1 mol eq. GSH; (**b**) the production of H_2_O_2_ before and 30 and 60 min after the oxidation of GSH. The data are mean ± SEM from 3 experiments (* *p* < 0.05).

Previously, Ito et al. [29] reported that RD oligomers prepared by the tyrosinase-catalyzed oxidation of RD have a strong pro-oxidant activity. In another study, Ito et al. [22] reported that the pro-oxidant activity of RES-oligomers also contributes to cytotoxicity, although it is weaker than that of RD oligomers. Therefore, in the present study, it was investigated whether the EQ-oligomer, which is an oligomer oxidation product of EQ **1**, has a pro-oxidant activity. EQ-oligomers were prepared by oxidizing 1 mM EQ **1** with tyrosinase (100 U/mL) for 120 min at pH 7.4. The EQ-oligomer was exposed to 1 mol eq. GSH and was followed for up to 60 min. Then, the remaining levels of GSH and GSSG were analyzed using 3,5-di-*tert.*-butyl-1,2-benzoquinone (DBBQ) with a specific HPLC method [17,37]. GSH levels were reduced by ca. 60% during the 60 min incubation period with EQ-oligomers (Figure 6a). Most of the GSH was oxidized to GSSG.

Next, we examined whether H_2_O_2_ was produced during the oxidation of GSH by these oligomers. As shown in the Figure 6b, 21 µM H_2_O_2_ was produced from the EQ-oligomer during the 60 min reaction with GSH (11 µM in the control).

## 3. Discussion

Topical application of EQ **1** is generally considered to be more beneficial than harmful to the skin [12]. Niwa et al. [30] recently reported that racemic EQ **1** inhibits mushroom tyrosinase in vitro. It is also known that daidzein and EQ **1** decrease the expression of tyrosinase, tyrosinase-related protein-1 (TRP-1), and tyrosinase-related protein-2 (TRP-2), thereby blocking melanin production in α-MSH-stimulated B16 melanoma cells [38]. However, in this study, it was shown that tyrosinase can effectively oxidize EQ **1** to produce two EQs with a mono-quinone skeleton (A and B) and one EQ with a di-quinone (C), all of which are highly reactive. The high reactivity is illustrated by their rapid decay (Figure 2) and the production of 3′-hydroxy-EQ **2** and 6-hydroxy-EQ **3** as mono-catechols and 6,3′-dihydroxy-EQ **4** as a di-catechol (Figure 1). EQ **1** is well known as a substrate for P450 enzymes. Rüfer et al. [33] reported that the main metabolites of (±)-EQ **1** in rat and human microsomal metabolisms are 3′-hydroxy-EQ **2** and 6-hydroxy-EQ **3**, but 6,3′-dihydroxy-EQ **4** was not identified. On the other hand, DiCys-EQ-catechol and DiGS-EQ-catechol **8** as the major metabolites of EQ **1** in human tyrosinase-transfected cells were identified. These results show that those metabolites are produced through EQ-quinone-C (Figure 1).

The reaction of EQ **1** with NAC in the presence of tyrosinase afforded 5′-monoNAC-3′-hydroxy-EQ **5** and 5,5′-diNAC-6,3′-dihydroxy-EQ **6**. Although the isolation of the CySH adducts was difficult because of their instability, two GSH adducts, 5′-monoGS-3′-EQ **7** and 5,5′-diGS-6,3′-dihydroxy-EQ **8**, using 3′-hydroxy-EQ **2** as a source of EQ-quinone, were isolated. These results suggest that EQ-quinone could be cytotoxic to melanocytes due to the binding of EQ-quinones to thiol proteins. Although not as potent as the RD-oligomer [17], the pro-oxidant activity of the EQ-oligomer may contribute to its higher cytotoxicity than the RES-oligomer according to the reduction in GSH levels [22]. Similarly, the production of H_2_O_2_ from the EQ-oligomer was less than that from the RD-oligomer [17] but more than that from the RES-oligomer [22]. We also examined whether EQ **1** was oxidized to EQ-quinone in T293 cells transfected with human tyrosinase. EQ **1** was metabolized in a dose-dependent manner to DiCys-EQ-catechol and DiGS-EQ-catechol **8** as major metabolites. Those metabolites were then released into the medium, and about 2% of the EQ **1** present in the medium was metabolized in the cells and released into the medium as Cys and GSH adducts during the 2 h incubation. The predominant production of di-adducts is consistent with the biochemical experiments. As EQ **1** is defined as a di-phenol with two *p*-substituted phenol structures, it was confirmed that EQ **1** can be oxidized to the corresponding di-o-quinone, indicating that it is a good substrate for not only mushroom tyrosinase but also human tyrosinase. These situations are similar to those observed with RD and RES being a good inhibitor as well as a good substrate of tyrosinase [14,15,16,22,27], including human tyrosinase [39]. Di-o-quinone generated by tyrosinase oxidation of EQ **1** bound to thiols to form di-adducts, which was confirmed in the tyrosinase-transfected cells.

In this study, mono- and di-adducts of EQ-quinones with thiol compounds in the presence of tyrosinase were obtained. As far as we are aware, this is the first study in which di-phenol produced di-o-quinone, and both were involved in adduct formation. This high reactivity of EQ-quinones would lead to cytotoxicity by inactivating the SH enzymes and by unfolding SH proteins causing endoplasmic reticulum (ER) stress. In this connection, it is known that RD induces the ER stress response and apoptosis in melanocytes [14,40]. 

## 4. Materials and Methods

### 4.1. Materials

(*S*)-(−)-Equol (7-hydroxy-3-(4′-hydroxyphenyl)-chroman) (EQ) **1** was purchased from Daicel Corporation (Osaka, Japan). Tyrosinase (from mushrooms, specific activity 2687 U/mg), L-cysteine (CySH), reduced glutathione (GSH), oxidized glutathione (GSSG), *N*-acetyl-L-cysteine (NAC), bovine serum albumin (BSA), horseradish peroxidase, Dulbecco’s Modified Eagle’s Medium (DMEM) containing 10% fetal bovine serum, and Ampliflu™ Red reagent (10-acetyl-3,7-dihydroxyphenoxazine) were purchased from Sigma-Aldrich (St. Louis, MO, USA). H_2_O_2_ was purchased from Mitsubishi Gas Chemical Co., Ltd. (Tokyo, Japan). Perchloric acid (HClO_4_) was purchased from Katayama Chemical Industries Co., Ltd. (Osaka, Japan). Formic acid (HCOOH), ethanol (HPLC grade), methanol (HPLC grade), ethyl acetate, NaBH_4_, 3,5-di-*tert.*-butyl-1,2-benzoquinone (DBBQ), L-ascorbic acid (AA), and 2-mercaptoethanol (thioglycol) were from FUJIFILM Wako Pure Chemical Corporation (Osaka, Japan). The highest purity Milli-Q water (Milli-Q Advantage, Merck Millipore Co., Tokyo, Japan) was used throughout this study to avoid contamination with metal ions. *N*-ethylmaleimide (NEM) was from Tokyo Chemical Industries (Tokyo, Japan). NEM-modified BSA was prepared by reacting 0.01 mmol BSA with 0.3 mmol NEM in 50 mM sodium phosphate buffer, pH 6.8 (10 mL) at 37 °C for 60 min. The NEM-modified BSA was mixed with 40 mL cold ethanol and precipitated by centrifugation, washed three times with 20 mL cold ethanol, and dissolved in the pH 6.8 buffer. The concentration of NEM-modified BSA was estimated from previous data to be 0.51 mM based on UV spectrophotometry at 280 nm.

### 4.2. Instruments

A HPLC system was used to follow the course of tyrosinase oxidation. It comprised an analytical UV/VIS detector, a JASCO pump (JASCO Co., Tokyo, Japan), a C18 column (Capcell Pak MG; 4.6 × 250 mm; 5 µm particle size, Osaka Soda, Osaka, Japan), and a JASCO UV/visible detector (JASCO Co., Tokyo, Japan). The mobile phase was 0.4 M HCOOH: methanol, 60:40 (*v/v*). Analyses were performed at 50 °C at a flow rate of 0.7 mL/min. For preparative separation, a C18 preparative column (Capcell Pak MG; 20 × 250 mm; 5 µm particle size, Osaka Soda, Osaka, Japan) was used at a flow rate of 7.0 mL/min with the mobile phase (0.4 M HCOOH: methanol, 50:50 (*v/v*)) at 45 °C. A Shiseido electrochemical detector (Shiseido, Tokyo, Japan) was also used to analyze low concentrations of metabolites, as electrochemical detection is >10-times more sensitive than UV/visible detection.

Nuclear magnetic resonance (NMR, 400 MHz for ^1^H) spectra were obtained using a Bruker AVANCE 400 spectrometer (Billerica, MA, USA). High-resolution mass spectra were obtained using a 6220 TOF mass spectrometer (mode: electrospray ionization—time-of-flight, negative; ESI(−) or positive; ESI (+)-TOF) (Agilent Technologies, Santa Clara, CA, USA).

For measurements of H_2_O_2_, the maximum absorption wavelength of 571 nm possessed by red fluorescent resorufin in which Ampliflu^TM^ Red was oxidized was used. UV/visible spectra were measured with a JASCO V-630 UV-VIS spectrophotometer (JASCO Co., Tokyo, Japan).

### 4.3. Oxidation of EQ **1** by Tyrosinase in the Absence or Presence of L-Ascorbic Acid or a Thiol

According to the method described by Ito et al. [22], a solution (2 mL) of 100 µM EQ **1** was oxidized by 50 U/mL tyrosinase at 37 °C in 50 mM sodium phosphate buffer (pH 6.8 or 5.3). Changes in absorption spectra were periodically followed for 60 min. The oxidation was also carried out in the presence of 1 mM AA or 0.3 mM NAC, CySH, or GSH. For spectrophotometric analysis, the reference cell contained the same concentrations of buffer, tyrosinase, and AA or a thiol. For HPLC analysis, the reaction was stopped by adding a 200 µL aliquot to 200 µL 0.8 M HClO_4_ or to 20 µL 10% NaBH_4_ (20 µL) followed by 180 µL 0.8 M HClO_4_.

### 4.4. Isolation of EQ-Catechols

According to the method described by Ito et al. [22], a solution of EQ **1** (24.23 mg, 0.1 mmol) and AA (176.2 mg, 1.0 mmol) in ethanol (2 mL) was mixed with 98 mL 50 mM sodium phosphate buffer (pH 6.8). The mixture was vigorously shaken at 37 °C to which tyrosinase (20,000 U) in 1 mL buffer was added. After 20 min of oxidation, 2 mL 6 M HCl was added to stop the oxidation, and the mixture was extracted twice with 100 mL ethyl acetate. After evaporation of ethyl acetate under reduced pressure, the residue was dissolved in HPLC elution buffer and was subjected to preparative HPLC. After drying each eluate, 6.85 mg (25% yield) of 6,3′-dihydroxy-EQ **4** (HPLC purity 91%), 3.00 mg (12%) of 6-hydroxy-EQ **3** (HPLC purity 91%), and 6.34 mg (25%) of 3′-hydroxy-EQ **2** (HPLC purity 100%) were obtained.

#### Hydroxy-EQ (EQ-catechol-A, **2**), 6-hydroxy-EQ (EQ-catechol-B, **3**), and 6,3′-dihydroxy-EQ (EQ-catechol-C, **4**)

The ^1^H NMR spectra of 3′-hydroxy-EQ **2**, 6-hydroxy-EQ **3**, and 6,3′-dihydroxy-EQ **4** are shown in Figure S1b–d, respectively. High-resolution ESI-TOF MS analysis of 3′-hydroxy-EQ **2**, 6-hydroxy-EQ **3**, and 6,3′-dihydroxy-EQ **4** gave pseudo-molecular ion peaks at *m/z* 257 ([M − H]^−^), *m/z* 257 ([M − H]^−^), and *m/z* 273 ([M − H]^−^), respectively (Figure S2a–c). 3′-hydroxy-EQ **2** and 6-hydroxy-EQ **3**: high-resolution MS 257.0829 and 257.0814, calculated for C_15_H_13_O_4_, 257.0819, respectively. 6,3′-dihydroxy-EQ **4**: high-resolution MS 273.0765, calculated for C_15_H_13_O_5_, 273.0768 (Figure S2a–c, respectively).

### 4.5. Isolation of MonoNAC-EQ-Catechol **5** and DiNAC-EQ-Catechol **6**

According to the method described by Ito et al. [22], a solution of EQ **1** (21.8 mg, 0.09 mmol), 3′-hydroxy-EQ **2** (2.58 mg, 0.01 mmol), and NAC (65.2 mg, 4.0 mmol) in ethanol (2 mL) was mixed with 98 mL 50 mM sodium phosphate buffer (pH 6.8). The mixture was vigorously shaken at 37 °C to which tyrosinase (20,000 U) in 1 mL buffer was added. After 20 min of oxidation, 2 mL 6 M HCl was added to stop the oxidation, and the mixture was extracted twice with 100 mL ethyl acetate. After evaporation of ethyl acetate under reduced pressure, the residue was dissolved in HPLC elution buffer and was subjected to preparative HPLC. After drying each eluate, 2.83 mg (5% yield) DiNAC-EQ-catechol **6** (HPLC purity 96%) and 4.96 mg (12%) monoNAC-EQ-catechol **5** (HPLC purity 100%) were obtained.

#### 4.5.1. 5′-Mono-*S*-[(*N*-acetyl)cysteinyl]-3′-hydroxy-EQ (MonoNAC-EQ-catechol, **5**)

The ^1^H NMR spectrum of monoNAC-EQ-catechol **5** is shown in Figure S3a. ESI(−) MS: *m/z* 418 ([M − H]^−^). High-resolution ESI-TOF MS analysis of the compound gave pseudo-molecular ion peaks at *m/z* 418 ([M − H]^−^). High-resolution MS 418.0980, calculated for C_20_H_20_NO_7_S_1_, 418.0966 (Figure S4a).

#### 4.5.2. 5,5′-Di-*S*-[(*N*-acetyl)cysteinyl]-6,3′-dihydroxy-EQ (DiNAC-EQ-catechol, **6**)

The ^1^H NMR spectrum of DiNAC-EQ-catechol **6** is shown in Figure S3b. ESI(−) MS: *m/z* 595 ([M − H]^−^). High-resolution MS 595.1069, calculated for C_25_H_27_N_2_O_11_, 595.1062 (Figure S4b).

### 4.6. Adduct Formation of EQ-Quinones with CySH and GSH and Isolation of GSH Adducts

According to the method described by Ito et al. [22], a solution of 3′-hydroxy-EQ **2** (2.5 mg, 0.01 mmol) and CySH (4.9 mg, 0.04 mmol) or GSH (12.4 mg, 0.04 mmol) in 10 mL of 50 mM sodium phosphate buffer (pH 6.8) was vigorously shaken at 37 °C, to which tyrosinase (2000 U) in 1 mL of buffer was added. After 10 min of oxidation, 0.2 mL of 6 M HCl was added to stop the oxidation, and the mixture was evaporated to dryness under reduced pressure. The residue was dissolved in HPLC elution buffer and was subjected to preparative HPLC. The CySH adduct was rapidly oxidized and thus could not be isolated. Lyophilization of the faster eluting and slower eluting fractions from GSH gave 5′-monoGS-3′-EQ **7** (3.35 mg, 59%) and 5,5′-diGS-6,3′-dihydroxy-EQ **8** (0.27 mg, 5% yield). The ^1^H NMR and ^13^C NMR spectra of 5′-monoGS-3′-EQ **7** and the ^1^H NMR of 5,5′-diGS-6,3′-dihydroxy-EQ **8** are shown in Figure S5a–f, respectively. The structure of 5′-monoGS-3′-EQ **7** was assigned in detail using shift correlation 2D NMR (^1^H-^1^H correlation spectroscopy (COSY)-NMR), ^1^H-^13^C Heteronuclear single quantum correlation (HSQC)-NMR, and ^1^H-^13^C heteronuclear multiple-bond correlation (HMBC)-NMR) (Figure S5d–f). The ^1^H NMR spectrum of 5′-monoGS-3′-EQ **7** with the 2D double quantum filtered (DQF)-COSY method is shown in Figure S5d. The ^1^H and ^13^C NMR spectra of 5′-monoGS-3′-EQ **7** with the 2D HSQC method and the 2D HMBC method are shown in Figure S5e,f, respectively. High-resolution ESI-TOF MS analysis of 5′-monoGS-3′-EQ **7** gave pseudo-molecular ion peaks at *m/z* 586 ([M + Na]^+^) (Figure S6a). The positions of two GSH groups in the benzene ring of 5,5′-diGS-6,3′-dihydroxy-EQ **8** were assigned by the positions of three protons: 6.36 ppm (1H, s), 6.72 ppm (1H, d, J = 2.0 Hz), and 6.83 ppm (1H, d, J = 2.0 Hz) (Figure S5c). This pattern of the positions of three protons in the benzene ring was the same as that in 5,5′-diNAC-6,3′-dihydroxy-EQ **6** (Figure S3b): 6.34 ppm (1H, s), 6.69 ppm (1H, d, J = 2 Hz), and 6.85 ppm (1H, d, J = 2.0 Hz). High-resolution ESI-TOF MS analysis of the di-GSH adducts gave pseudo-molecular ion peaks at *m/z* 907 ([M + Na]^+^) (Figure S6b). The ^1^H NMR of EQ metabolites (**2**, **3**, **4**, **5**, and **6**) are summarized in Table S1. ^1^H and ^13^C NMR spectra of 5′-monoGS-3′-EQ **7** are summarized in Table S2. The number of GSH additions was confirmed by MS analysis. 5′-monoGS-3′-EQ **7**; ESI(+) MS: *m/z* 586 ([M + Na]^+^), high-resolution ESI-TOF MS 586.1466, calculated for C_25_H_29_N_3_NaO_10_S, 586.1466 (Figure S6a). 5,5′-diGS-6,3′-dihydroxy-EQ **8**; ESI(+) MS: *m/z* 907 ([M + Na]^+^), high-resolution MS 907.2099, calculated for C_35_H_44_N_6_NaO_17_S_2_, 907.2097 (Figure S6b).

### 4.7. Metabolism of EQ **1** in Tyrosinase-Transfected Cells

293T cells (0.75 × 10^6^) were transfected with the human tyrosinase gene. The expression plasmid pcDNA3.1 containing the coding sequence of human *TYR* gene was transiently transfected using Lipofectamine 2000 Reagent (Invitrogen, Carlsbad, CA, USA) according to the manufacturer’s protocol. The cells were then incubated in DMEM, and, 24 h later, the medium (0.75 mL) was replaced with medium containing 0.0, 0.05, 0.1, and 0.2 mM EQ **1.** The incubation was then continued for 2 h. Medium samples (0.18 mL) were deprotonated with 4 M HClO_4_ at 4 °C for 60 min. Cell samples (0.75 × 10^6^) were extracted with 0.4 M HClO_4_ by shaking vigorously for 60 min. After centrifugation at 10,000× *g* for 3 min, the supernatants were analyzed by HPLC as described below. 

Concentrations of EQ **1** remaining in the medium or taken up in cells were analyzed using a mobile phase of 0.4 M HCOOH: methanol = 50:50 (*v/v*) at 45 °C with a flow rate of 0.7 mL/min and a wavelength of 280 nm. EQ **1** appeared at 10.9 min under these conditions. Concentrations of EQ-quinone adducts of CySH and GSH in the medium or in cells were analyzed using a mobile phase of 0.4 M HCOOH: methanol = 70:30 (*v/v*) at 45 °C with a flow rate of 0.7 mL/min. To detect low levels of those adducts, an electrochemical detector with an applied voltage of 600 mV was used.

### 4.8. Pro-Oxidant Activity of the EQ **1** Oxidation Product, EQ-Oligomer

According to the method described by Ito et al. [22], the EQ-oligomer was prepared in 50 mM sodium phosphate buffer (pH 7.4) from EQ **1** (1 mM). Tyrosinase (200 U) was added to the 2 mL precursor solution, and the mixture was incubated at 37 °C for 120 min. Tyrosinase alone (100 U/mL) was used as a control. The melanin solutions (2 mL) were mixed with 10 mM GSH (200 µL, 1 mol eq.) and were incubated at 37 °C. At 0, 30, and 60 min reaction times, 100 µL aliquots were withdrawn and mixed with 0.4 M HClO_4_ (800 µL) to terminate the oxidation. GSH and GSSG in the oxidation mixtures were analyzed using the HPLC method described by Imai et al. [37]. The HPLC system was modified from the original conditions as follows: a mobile phase of 0.4 M HCOOH: methanol, 30:70 (*v/v*) was used with a UV detector at 294 nm and a column temperature of 45 °C.

According to the method described by Ito et al. [22], H_2_O_2_ was analyzed spectrophotometrically after dilution with the pH 7.4 buffer. Briefly, the oxidation mixture (20 µL) was diluted with pH 7.4 buffer (180 µL), and the diluted mixture (200 µL) was reacted with the chromogen Ampliflu™ Red reagent (200 µL) to form a red pigment with an absorption maximum at 568 nm [41], closely following the manufacturer’s instructions (Invitrogen, Tokyo, Japan). The mixture was left at room temperature for 10 min. Absorption spectra were measured between 450 and 650 nm.

### 4.9. Statistical Analyses

Student’s *t*-tests (two-tailed) were performed using Microsoft Excel for Mac (Japan Microsoft Co., Tokyo, Japan). A *p*-value of <0.05 was considered statistically significant.

## 5. Conclusions

EQ-quinones generated by the oxidation of EQ **1** in the presence of tyrosinase reacted with NAC, CySH, GSH, and BSA through the CySH residue. EQ **1** was also oxidized to EQ-quinones in cells expressing human tyrosinase. The EQ-oligomer can oxidize GSH to GSSG, indicating its pro-oxidant activity. These results suggest that EQ-quinones could be cytotoxic to melanocytes due to the binding of EQ-quinones to their proteins. Although EQ **1** has various beneficial effects on human health, it is considered necessary to use EQ **1** with the utmost care when applying it for cosmetic purposes.

## Supplementary Materials

Supplementary Materials can be found at https://www.mdpi.com/article/10.3390/ijms22179145/s1.

## Abbreviations

AA	L-Ascorbic acid
Ampliflu™ Red reagent	10-Acetyl-3,7-dihydroxyphenoxazine
BSA	Bovine serum albumin
COSY	Correlation spectroscopy
CySH	Cysteine
DBBQ	3,5-di-*tert.*-butyl-1,2-benzoquinone
DMEM	Dulbecco’s modified eagle’s medium
DQF	Double quantum filtered
DTNB	5.5′-Dithiobis (2-nitrobenzoic acid)
ER	Endoplasmic reticulum
EQ	Equol (7-hydroxy-3-(4′-hydroxyphenyl)-chroman)
ESI	Electrospray ionization
GSH	Glutathione
GSSG	Oxidized glutathione
HMBC	Heteronuclear multiple-bond correlation
HPLC	High-performance liquid chromatography
HSQC	Heteronuclear single quantum correlation
NAC	*N*-Acetyl-L-cysteine
NEM	*N*-Ethylmaleimide
NMR	Nuclear magnetic resonance
SOD	Superoxide dismutase
TOF MS	Time of flight mass spectrometry
TRP-1	Tyrosinase-related protein-1
TRP-2	Tyrosinase-related protein-2
UV	Ultraviolet
