# Supplementary material for: The Oxidation of Equol by Tyrosinase Produces a Unique Di-ortho-Quinone: Possible Implications for Melanocyte Toxicity
Source: Int J Mol Sci. 2021 Aug 24;22(17):9145. doi: 10.3390/ijms22179145 (PMC8431114; doi:10.3390/ijms22179145)
Supplement: Supplementary file 1 [file ijms-22-09145-s001.zip › Suppementary Materials_Equol_210819.pdf]

Supplementary Materials:

Figure S1. a) ^{1}H-NMR of (*S*)-EQ **1**

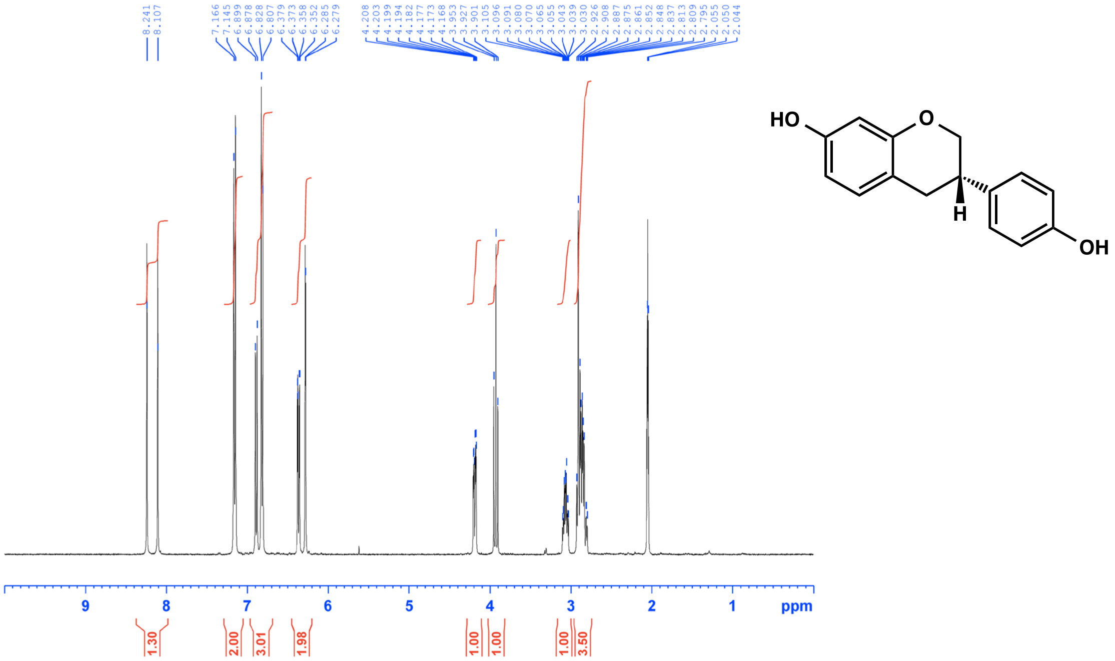

Figure S1. b) ^{1}H-NMR of 3’-hydroxy-EQ **2**

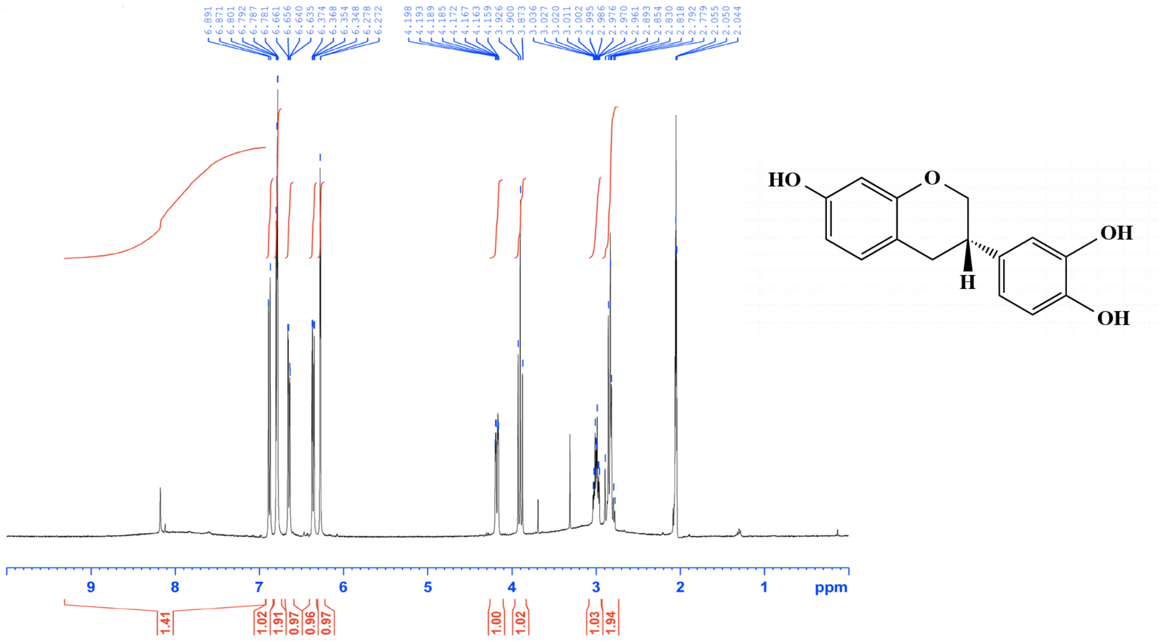

Figure S1. c) ^{1}H-NMR of 6-hydroxy-EQ **3**

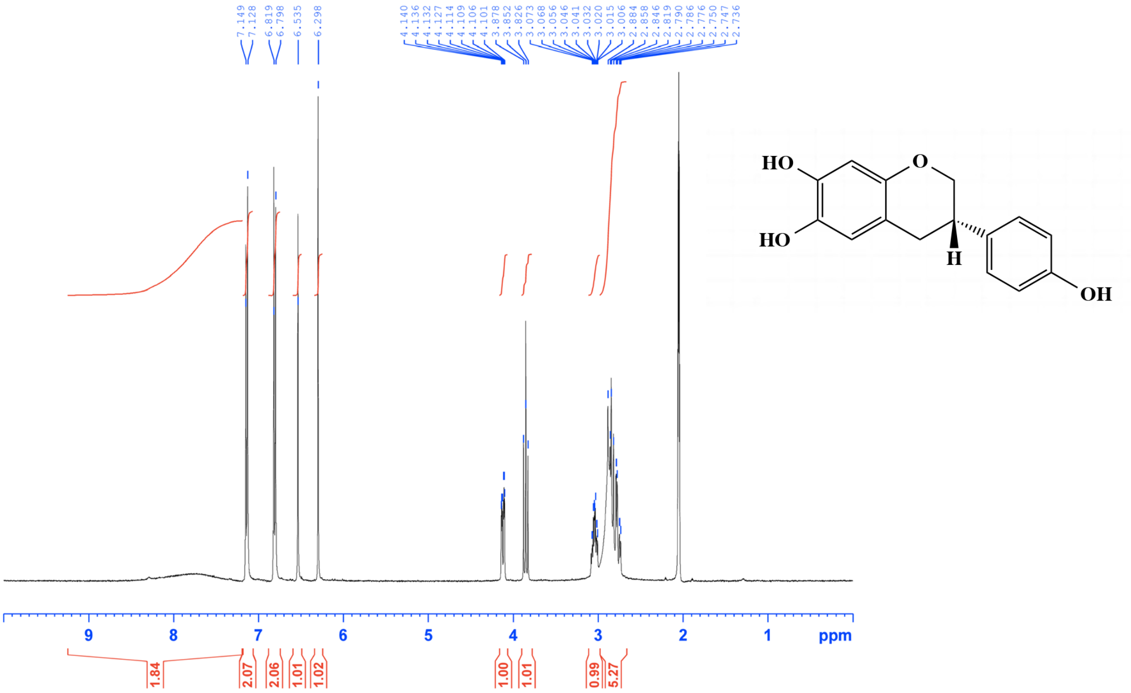

Figure S1. d) ^{1}H-NMR of 6.3'-dihydroxy-EQ **4**

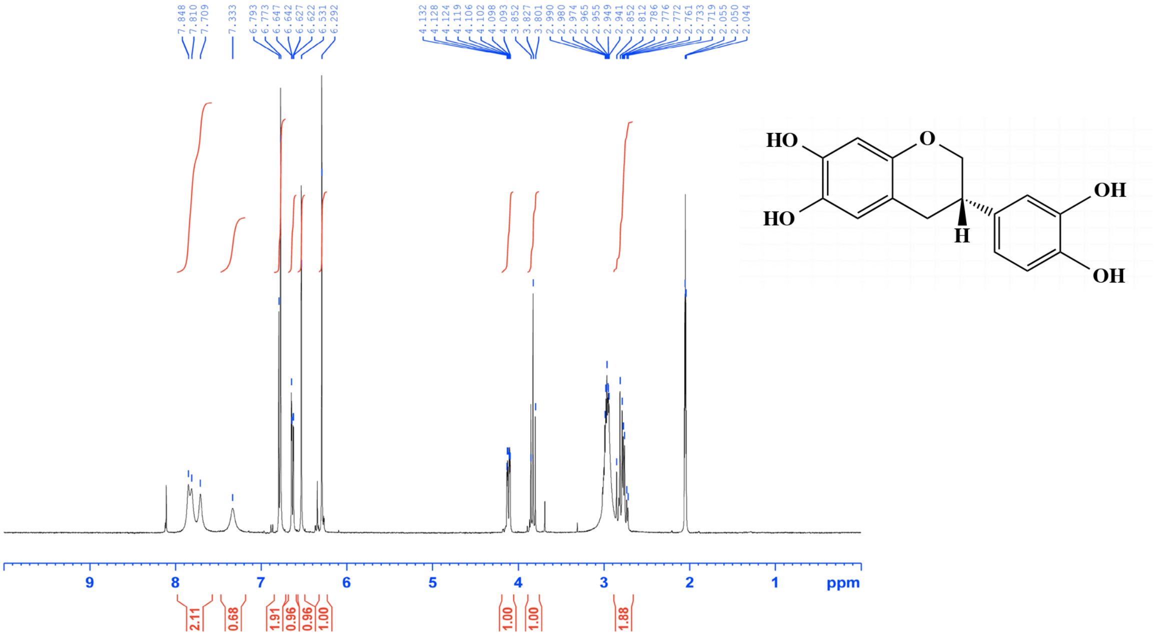

Figure S2. a) ESI TOF MS of 3’-hydroxy-EQ **2**

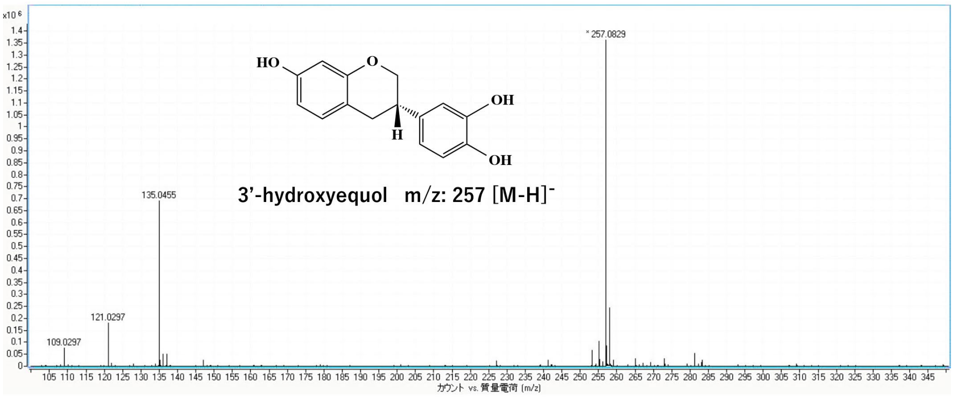

Figure S2. b) ESI TOF MS of 6-hydroxy-EQ **3**

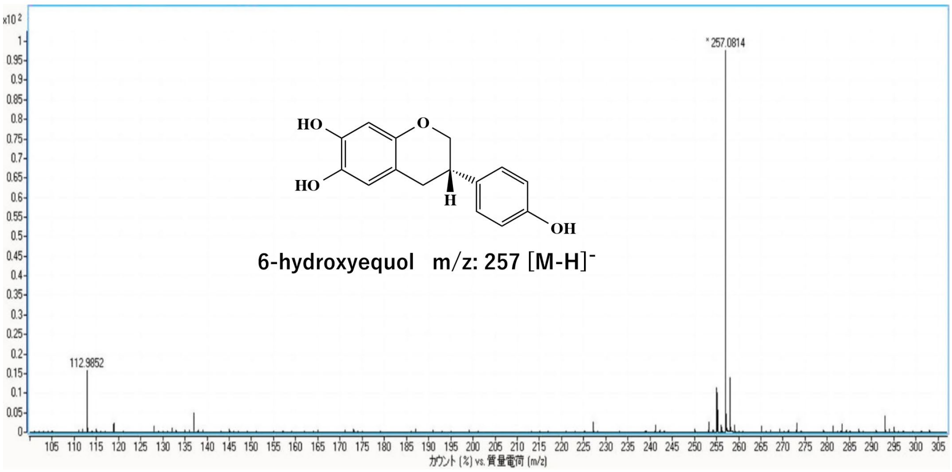

Figure S2. c) ESI TOF MS of 6,3'-dihydroxy-EQ **4**

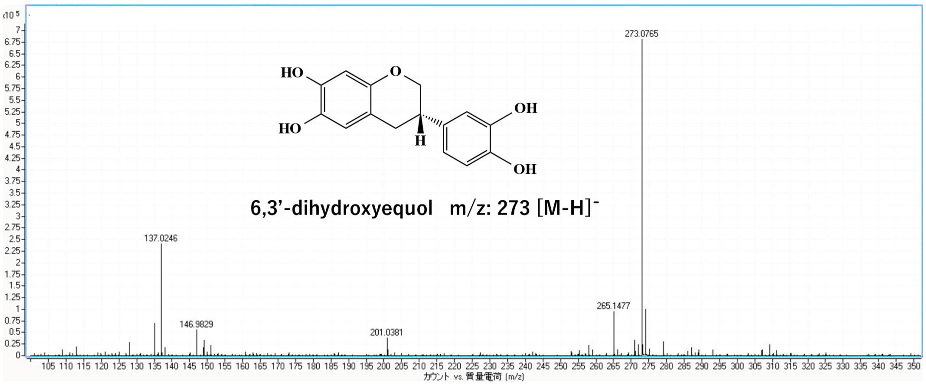

Figure S3. a) ^{1}H-NMR of 5'-monoNAc-3'-hydroxy-EQ **5**

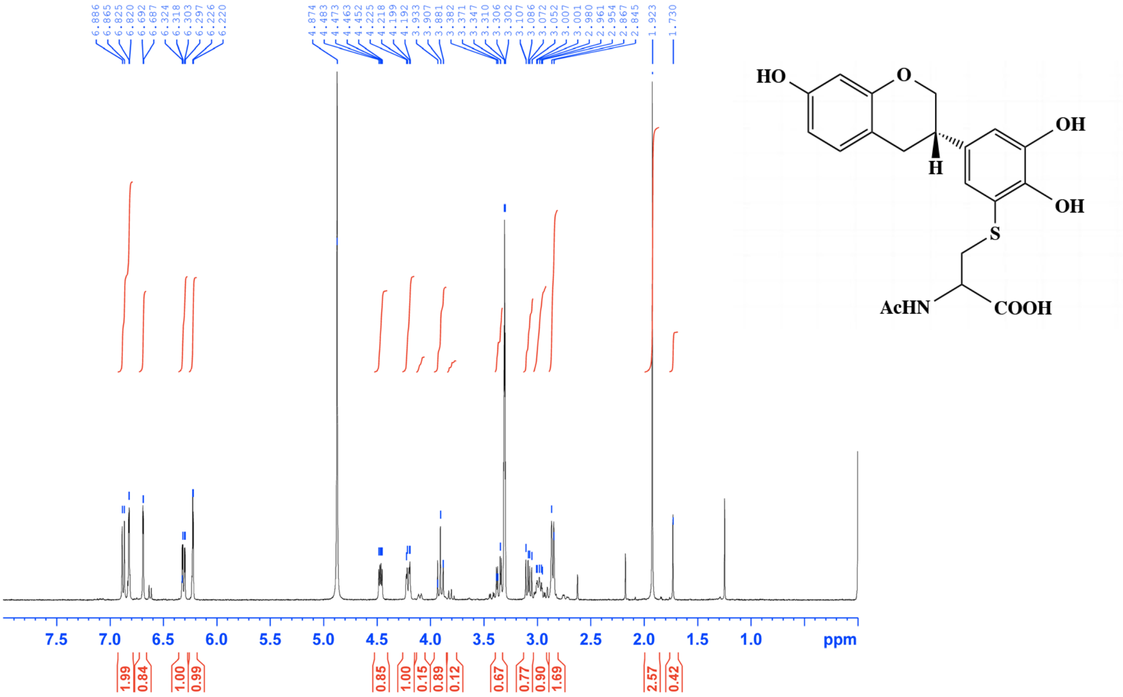

Figure S3. b) ^{1}H-NMR of 5,5'-diNAc-6,3'-dihydroxy-EQ **6**

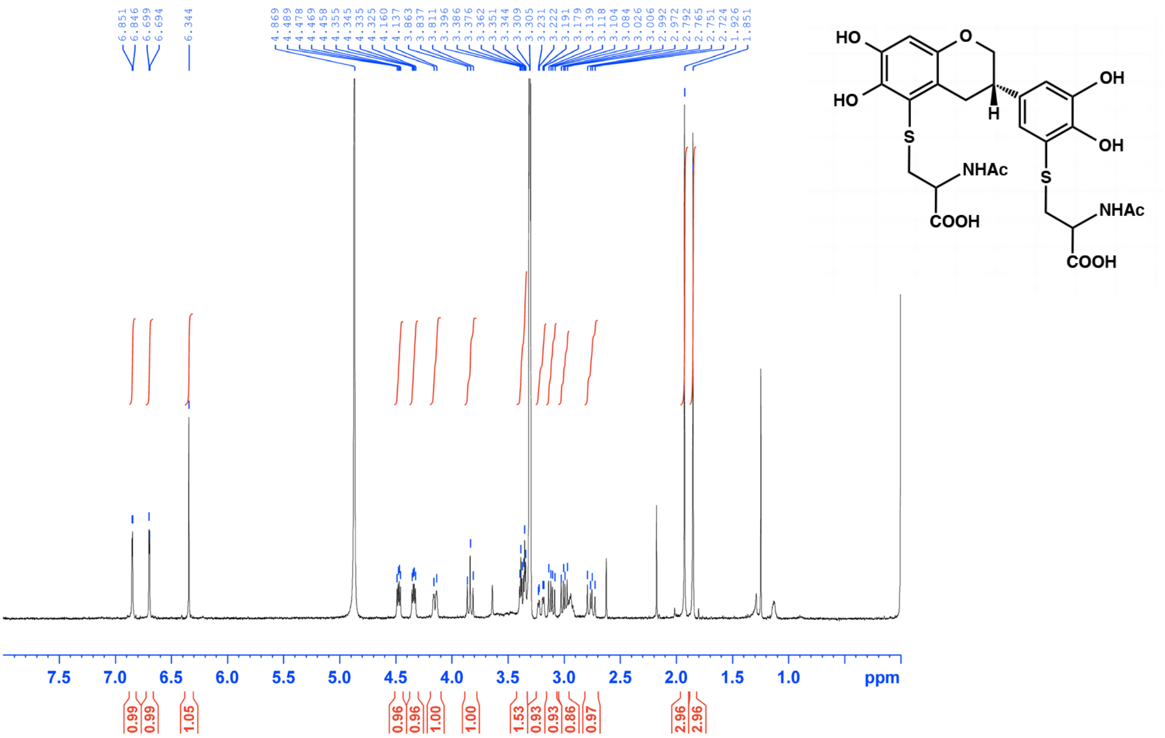

Figure S4. a) ESI TOF MS of 5'-monoNAc-3'-hydroxy-EQ **5**

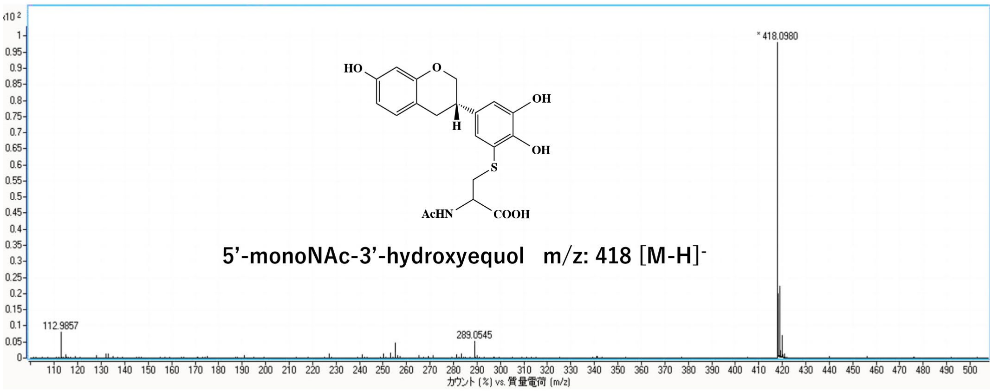

Figure S4. b) ESI TOF MS of 5,5'-diNAc-6,3'-dihydroxy-EQ **6**

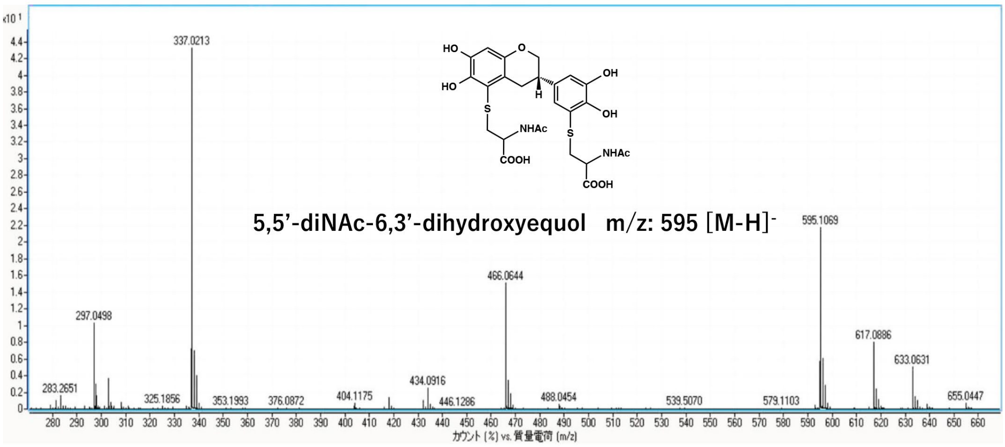

Figure S5. a) ^{1}H-NMR of 5’-monoGS-3’-EQ **7**

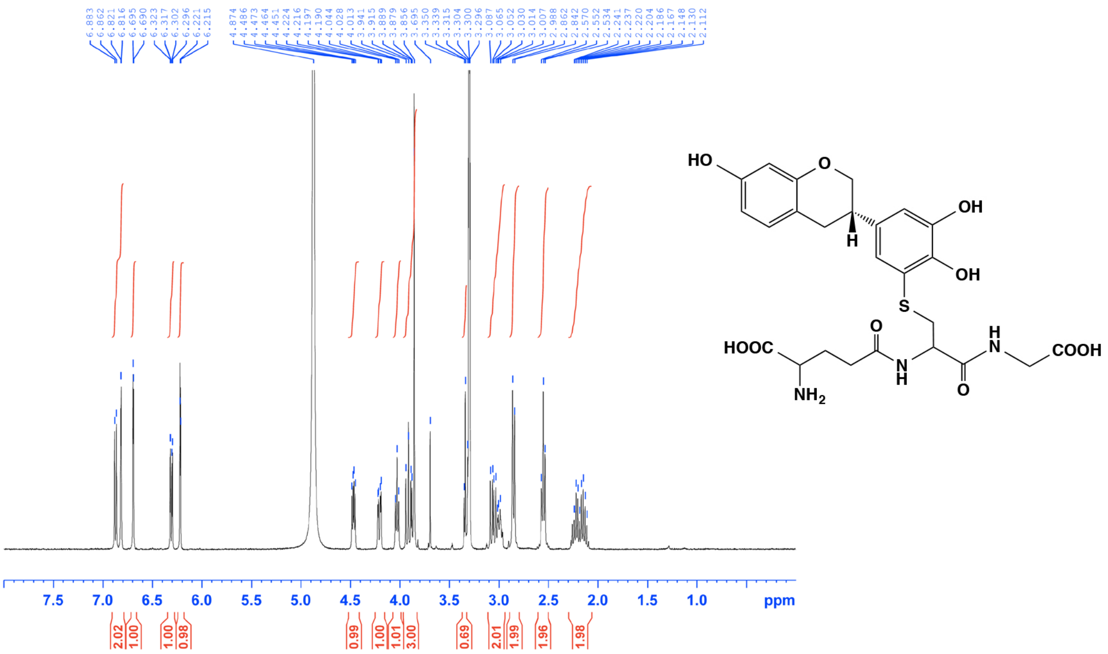

Figure S5. b) ^{13}C-NMR of 5’-monoGS-3’-EQ **7**

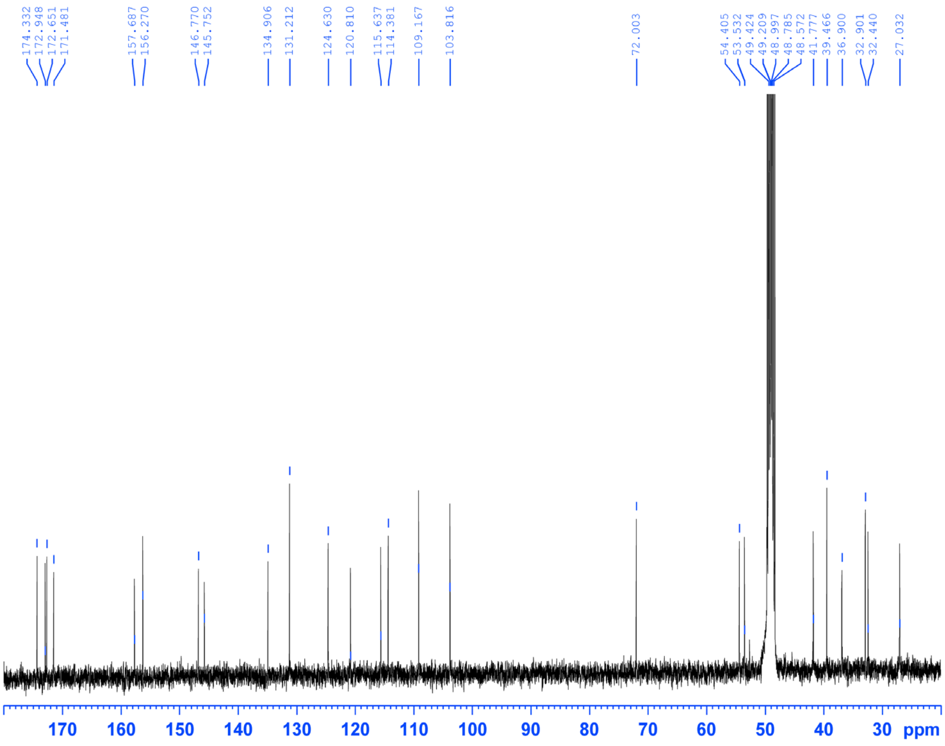

Figure S5. c) ^{1}H-NMR of 5,5'-diGS-6,3'-dihydroxy-EQ **8**

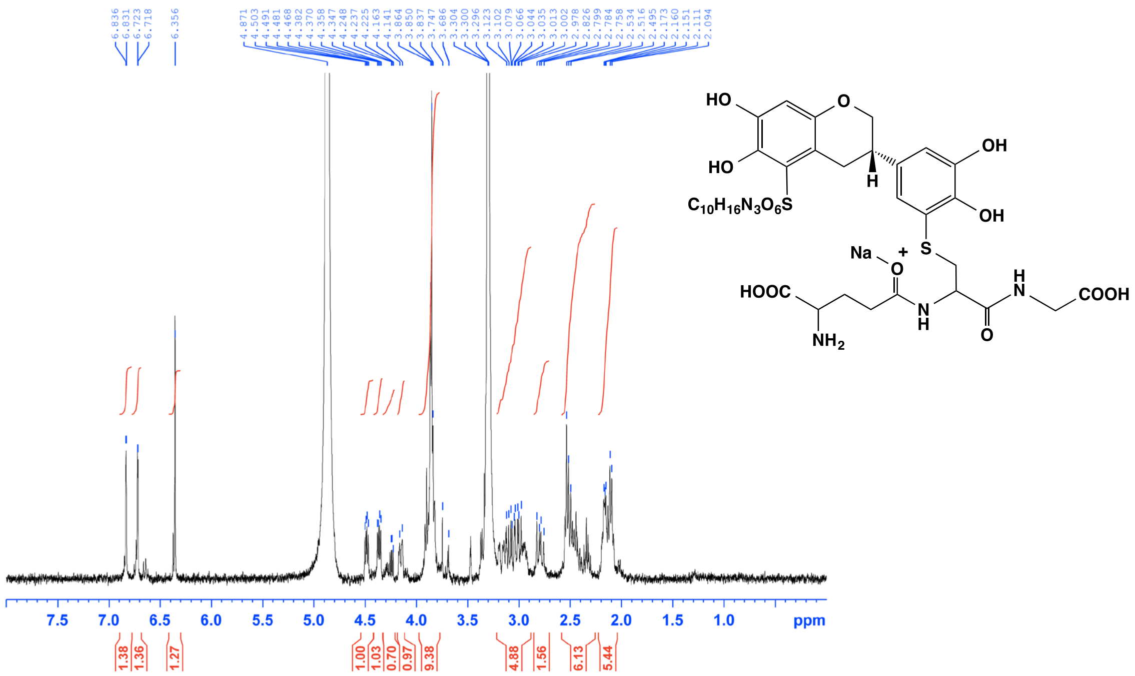

Figure S5. d) ^{1}H-^{1}H COSY-NMR of 5'-monoGS-3'-EQ **7**

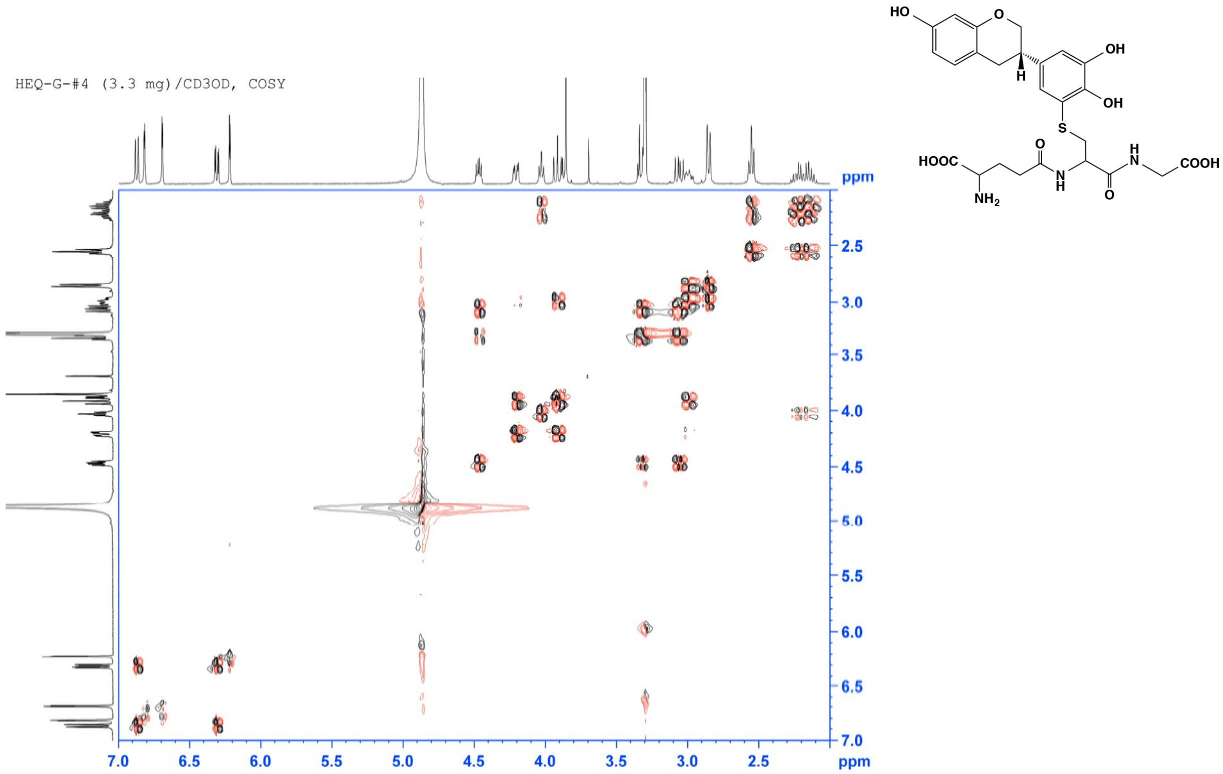

Figure S5. e) ^{1}H-^{13}C HSQC-NMR of 5'-monoGS-3'-EQ **7**

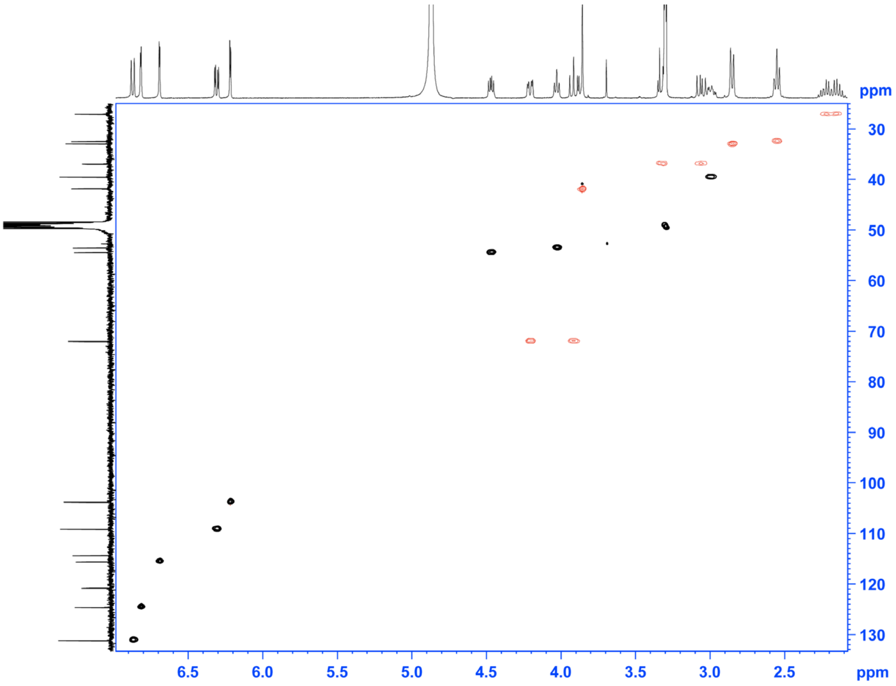

Figure S5. f) ^{1}H-^{13}C HMBC-NMR of 5'-monoGS-3'-EQ **7**

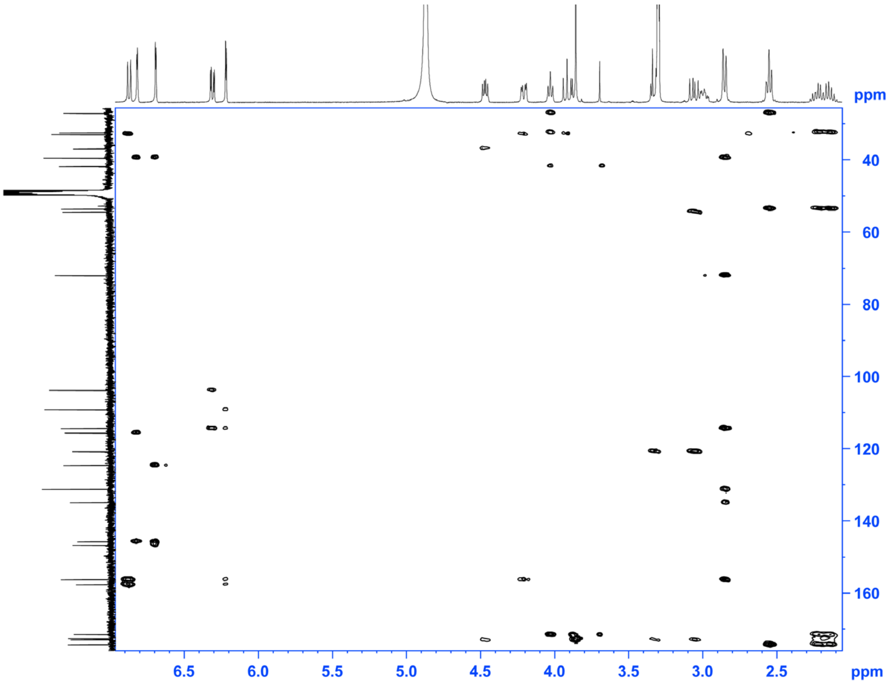

Figure S6. a) HR ESI-TOF MS of 5'-monoGS-3'-EQ **7**

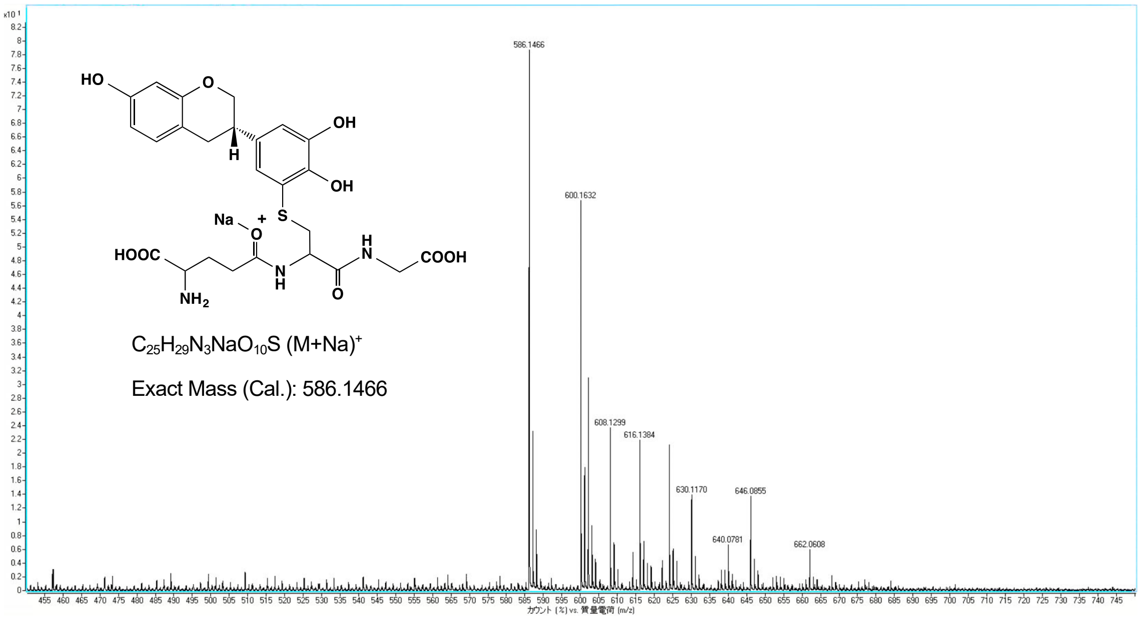

Figure S6. b) HR ESI-TOF MS of 5,5'diGS-6,3'-dihydroxy-EQ **8**

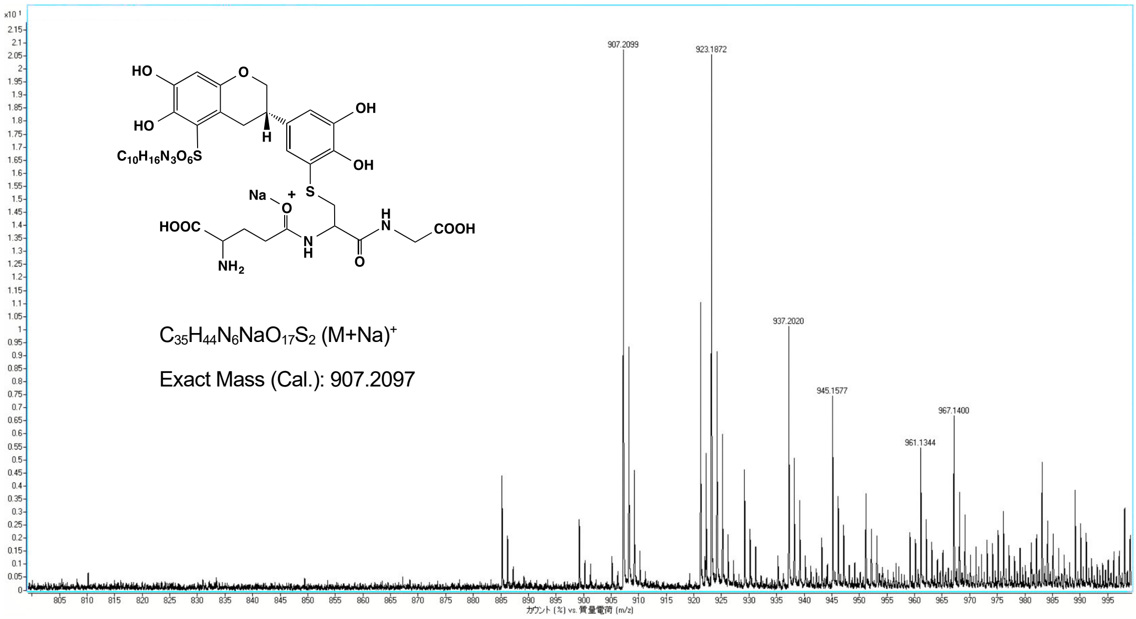

Table S1. Assignment of ^{1}H of EQ metabolites (**2**, **3**, **4**, **5**, and **6**) in comparison with those of EQ **1**.[a]

Position	EQ **1**	3'-hydroxy-EQ **2**	6-hydroxy-EQ **3**	6.3'-dihydroxy-EQ **4**	5'-monoNAc-3'-hydroxy-EQ **5**	5,5'-diNAc-6,3'-dihydroxy-EQ **6**
H-2	3.93 (1H, dd, J = 10.4, 10.4 Hz)	3.90 (1H, dd, J = 10.4. 10.4 Hz)	3.85 (1H, dd, J = 10.4, 10.4 Hz)	3.83 (1H, dd, J = 10.4, 10.4 Hz)	3.91 (1H, dd, J = 10.4, 10.4 Hz)	3.84 (1H, dd, J = 10.4, 10.4 Hz)
	4.20 (1H, m)	4.19 (1H, m)	4.12 (1H, m)	4.11 (1H, m)	4.20 (1H, m)	4.15 (1H, m)
H-3	3.08 (1H, m)	3.00 (1H, m)	3.04 (1H, m)	2.97 (1H, m)	2.97 (1H, m)	3.20 (1H, m)
H-4	2.85 (2H, m)	2.82 (2H, m)	2.79 (2H, m)	2.77 (2H, m)	3.08 (1H, dd, J = 8.0, 14.0 Hz)	3.11 (1H, dd, J = 8.4, 14.0 Hz)
					3.37 (1H, dd, J = 4.4, 14.0 Hz)	3.37 (1H, m)
H-5	6.88 (1H, d, J = 8.4 Hz)	6.88 (1H, d, J = 8.0 Hz)	6.53 (1H, s)	6.53 (1H, s)	6.87 (1H, d, J = 8.4 Hz)	
H-6	6.36 (1H, dd, J = 8.4, 2.4 Hz)	6.36 (1H, dd, J = 8.0, 2.4 Hz)			6.31 (1H, dd, J = 2.4, 8.4 Hz)	
H-8	6.28 (1H, d, J = 2.4 Hz)	6.27 (1H, d, J = 2.4 Hz)	6.30 (1H, s)	6.29 (1H, s)	6.22 (1H, d, J = 2.4 Hz)	6.34 (1H, s)
H-2'	7.15 (1H, d, J = 8.4 Hz)	6.78 (1H, d, J = 2.0 Hz)	7.14 (1H, d, J = 8.4 Hz)	6.77 (1H, d, J = 2.0 Hz)	6.69 (1H, d, J = 2.0 Hz)	6.69 (1H, d, J = 2.0 Hz)
H-3'	6.81 (1H, d, J = 8.4 Hz)		6.80 (1H, d, J = 8.4 Hz)			
H-5'	6.81 (1H, d, J = 8.4 Hz)	6.65 (1H, d, J = 8.0 Hz)	6.80 (1H, d, J = 8.4 Hz)	6.63 (1H, d, J = 8.0 Hz)		
H-6'	7.15 (1H, d, J = 8.4 Hz)	6.79 (1H, dd, J = 8.0, 2.0 Hz)	7.14 (1H, d, J = 8.4 Hz)	6.78 (1H, dd, J = 8.0, 2.0 Hz)	6.82 (1H, d, J = 2.0 Hz)	6.85 (1H, d, J = 2.0 Hz)
OH	8.11 (s, 1H), 8.24 (s, 1H)					
$SCH_2C\underline{H}$(H)					4.47 (1H, m)	4.34 (1H, m), 4.47 (1H, m)
SCH_2(H)					2.85 (2H, m)	2.76 (1H, m), 3.00 (1H, m)
$COCH_3$(H)					1.92 (3H, s)	1.85 (3H, s), 1.93 (3H, s)

[a] The spectra of compounds (**1**, **2**, **3** and **4**) were taken in actone-d6. The spectra of compounds (**5** and **6**) were taken in CD_3OD. Chemical shifts are shown in ppm relative to the solvent signal at 2.05 ppm (acetone-d6) or 3.30 ppm (CD_3OD) in ^{1}H NMR, respectively.

Table S2. Assignment of ^{1}H and ^{13}C NMR spectra of 5’-monoGS-3’-EQ **7**.

Position	5’-monoGS-3’-EQ **7**	Position	5’-monoGS-3’-EQ **7**	Position	5’-monoGS-3’-EQ **7**
H-2	3.91 (1H, t, J = 10.4 Hz)	C-2	72.0	a (C)	171.5
	4.21 (1H, m)	C-3	39.5	b (C)	53.5
H-3	2.99 (1H, m)	C-4	36.9	c (C)	27.0
H-4	3.07 (1H, m)	C-4a	114.4	d (C)	32.4
	3.34 (1H, m)	C-5	131.2	e (C)	174.3
H-5	6.87 (1H, d, J = 8.4 Hz)	C-6	109.2	f (C)	54.4
H-6	6.31 (1H, dd, J = 2.4, 8.4 Hz)	C-7	156.3	g (C)	32.9
H-8	6.22 (1H, d, J = 2.4 Hz)	C-8	103.8	h (C)	172.9
H-2’	6.69 (1H, d, J = 2.0 Hz)	C-8a	157.7	i (C)	41.8
H-3’		C-1’	134.9	j (C)	172.7
H-5’		C-2’	115.6		
H-6’	6.82 (1H, d, J = 2.0 Hz)	C-3’	146.8		
b (H)	4.03 (1H, t, J = 6 Hz)	C-4’	145.8		
c (H)	2.11 - 2.24 (2H, m)	C-5’	120.8		
d (H)	2.55 (2H, m)	C-6’	124.6		
f (H)	4.47 (1H, dd, J = 5.2, 8.8 Hz)				
g (H)	2.85 (2H, m)				
i (H)	3.86 (2H, s)				

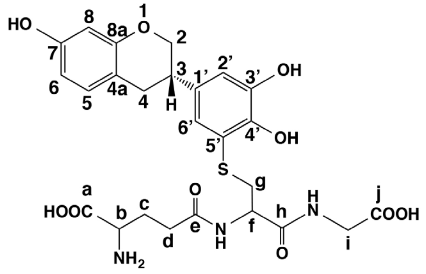

The spectra of 5’-monoGS-3’-EQ **7** was taken in CD_3OD. Chemical shifts are shown in ppm relative to the solvent signal at 3.30 ppm (CD_3OD) in ^{1}H NMR and at 49.00 ppm in ^{13}C NMR, respectively.
